# Dataset of anomalies and malicious acts in a cyber-physical subsystem

**DOI:** 10.1016/j.dib.2017.07.038

**Published:** 2017-07-20

**Authors:** Pedro Merino Laso, David Brosset, John Puentes

**Affiliations:** a Chair of Naval Cyber Defense, École Navale - CC 600, F29240 Brest Cedex 9, France; b Naval Academy Research Institute, École Navale - CC 600, F29240 Brest Cedex 9, France; c Institut Mines-Télécom Atlantique, Lab-STICC CNRS UMR 6285, Equipe DECIDE, F-29238 Brest, France

**Keywords:** Anomaly, Cyber-physical system, Sensor data, Systems security

## Abstract

This article presents a dataset produced to investigate how data and information quality estimations enable to detect aNomalies and malicious acts in cyber-physical systems. Data were acquired making use of a cyber-physical subsystem consisting of liquid containers for fuel or water, along with its automated control and data acquisition infrastructure. Described data consist of temporal series representing five operational scenarios – Normal, aNomalies, breakdown, sabotages, and cyber-attacks – corresponding to 15 different real situations. The dataset is publicly available in the .zip file published with the article, to investigate and compare faulty operation detection and characterization methods for cyber-physical systems.

**Specifications Table**

**Table:** 

Subject area	*Cyber-physical systems*
More specific subject area	*Anomaly detection and security*
Type of data	*Raw signal measurements directly collected from a liquid storage and distribution cyber-physical subsystem, composed by one ultrasound depth sensor, four discrete sensors, two pumps, and a communication network*
How data was acquired	*A personal computer sounder recorded all the signals in synchroNous automatic mode, scanning every 0.1 s the system's programmable logic controller (PLC)*
Data format	*Comma separated values (CSV) files*
Experimental factors	*Fifteen situations were recorded separately including – Normal, blocked measures, floating objects on the liquid's surface, sensor failure, denial of service, spoofing, wrong connection, and hit of the tanks with different intensities – to illustrate five factual operational scenarios*
Experimental features	*Relations between dysfunctional components of the cyber-physical subsystem, operational scenario, and systemic effects are represented*
Data source location	
Data accessibility	*The data are available with this article. To access it open the index.html file included in the published .zip file*

**Value of the data**

• The dataset represents realistic sensors signals of a cyber-physical subsystem impacted by actual risks like aNomalies, sabotages, system breakdown, and cyber-attacks.
• The dataset can be used to validate detection and characterization algorithms for operational surveillance and security applications in cyber-physical systems.
• Included aNomalies and malicious acts can be studied to compare detection and characterization approaches for decision support.
• The dataset can be used to examine algorithms that assess data alteration and service degradation.

## 1 Data

The dataset contains 15 files of temporal series that represent 15 different situations related to 5 operational scenarios. Files’ duration varies depending on the situation and dysfunctional component. Accordingly, affected components are two types of depth sensor, the underlying network, or the whole subsystem. These situations can be wrongly understood by a decision maker, or only identified for instance after the malicious act was accomplished. Since wrongly managed situations might have significant adverse operational costs, it is critical to detect and analyze in real time such events. Datasets covering such situations are currently rare, because of the complexity to acquire data from cyber-physical systems. In our case, the principle of reusable experimental platform [1] was applied, to collect diverse datasets for monitoring [2] and categorization of aNomalies [3].

## 2 Experimental design, materials and methods

**Fig. 1:**
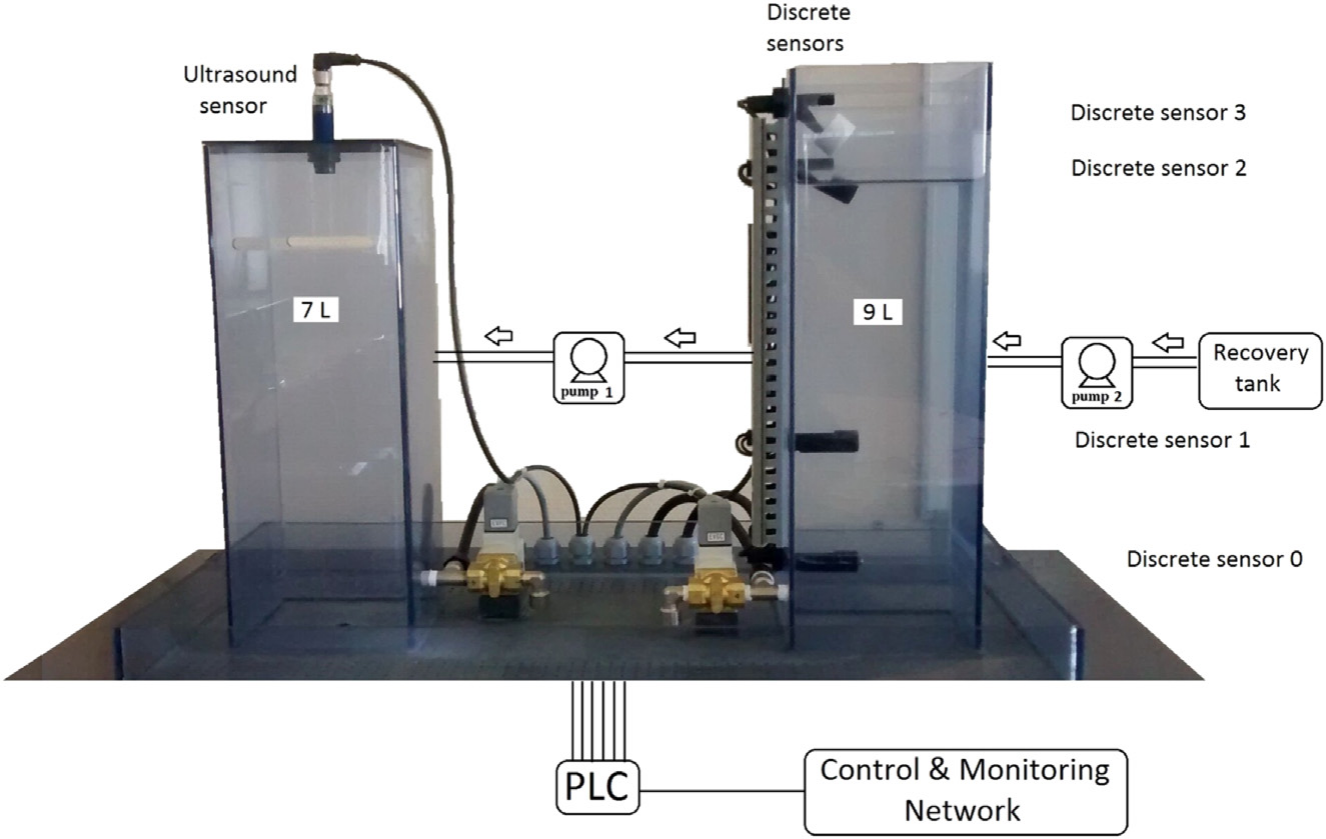
Platform of the used cyber-physical subsystem.

**Fig. 2:**
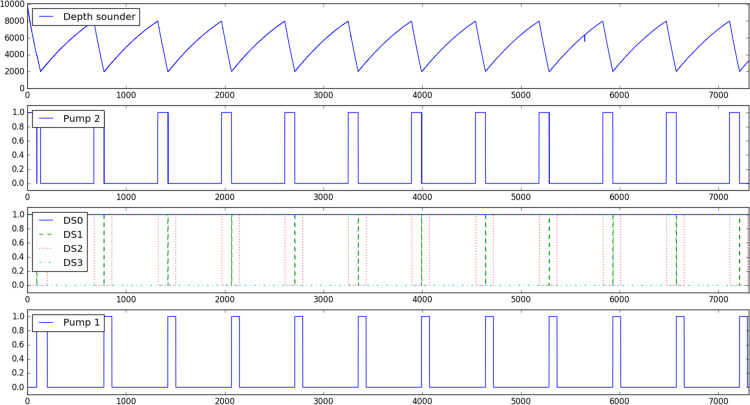
Set example of recorded temporal series for the Normal scenario (abscissas correspond to time in seconds). From top to down: periodic liquid filling and emptying of the main tank as indicated by the ultrasound depth sensor; activation of pump 2 to fill the second tank; state of the four discrete sensors in the second tank; activation of pump 1 to fill the main tank.

Two tanks of different volumes that function as storage and distribution device for water or fuel, one ultrasound depth sensor, four discrete sensors, and two pumps, were used to acquire the dataset (Fig. 1). A computer controlled the system with a PLC connected to a monitoring network. The ultrasound depth sensor on the main tank (volume of 7 L) was calibrated relating the tank dimensions to 10,000 equidistant depth steps (0 corresponds to the full tank and 10,000 to the empty tank). Fig. 2 shows the tracked filling and emptying of the main tank. The four floating discrete sensors in the second tank (volume of 9 L), measured levels of liquid corresponding to four volumes: 1.25 L, 3.35 L, 8 L, and 9 L.

**Fig. 3:**
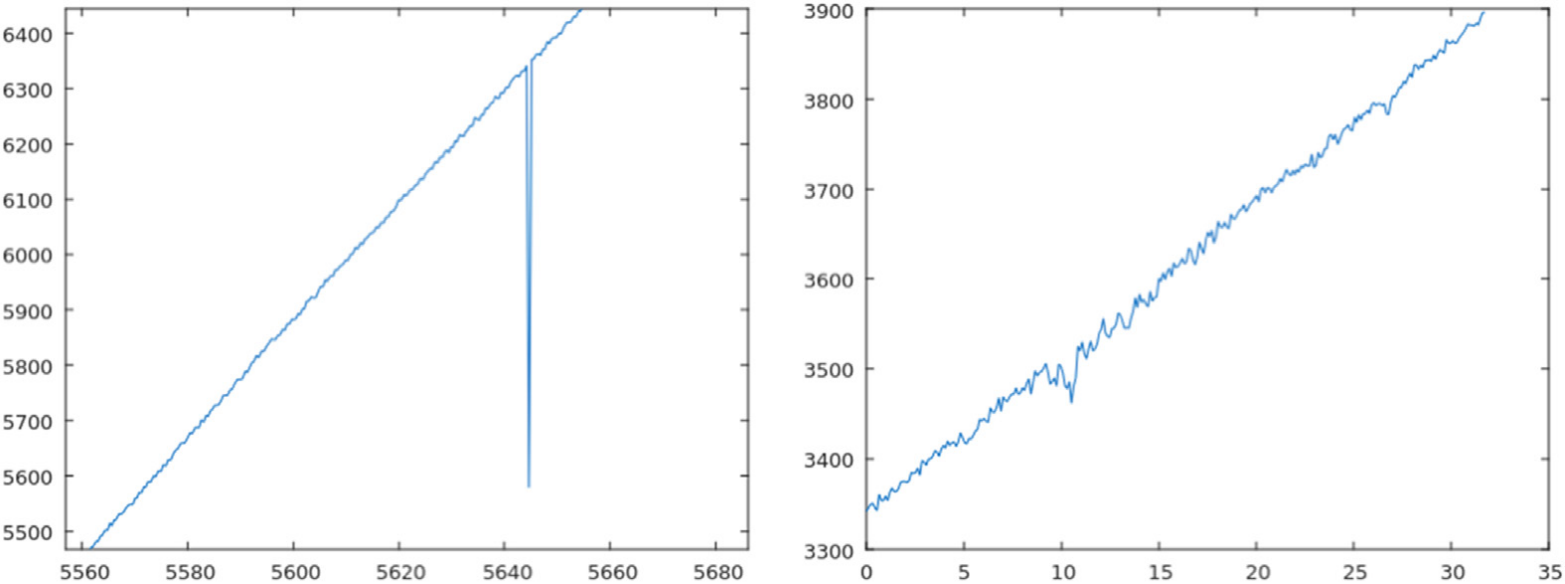
Left: Environmental aNomaly detected in the reference scenario (No. 1). Right: Noise produced by a plastic film over the sensor (No. 2).

**Fig. 4:**
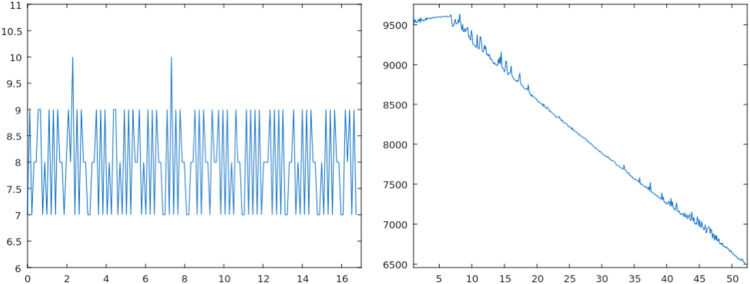
Left: Blocked sensor (No. 3). Right: Perturbations produced by floating objects (No. 5).

**Fig. 5:**
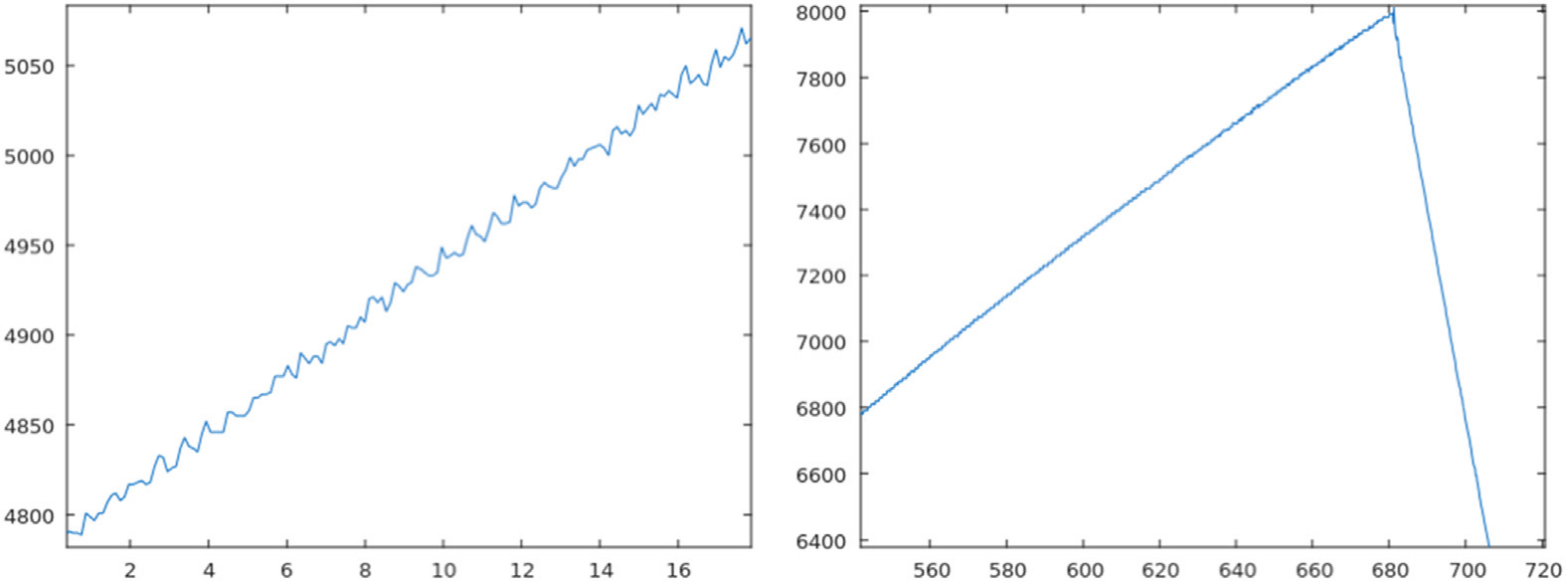
Left: Signal of the wet sensor (No. 7). Right: Perturbations caused while hitting the tanks (No. 14).

All signals *–* ultrasound depth sensor, pump 1, pump 2, and the four discrete level sensors *–* were acquired synchroNously for every situation described in Table 1, independently of the affected component, operational scenario, and duration. The Normal scenario without aNomalies serves as reference. Nine situations focus on the ultrasound depth sensor, since its high resolution makes it more sensitive to show aNomalies (No. 2, No. 3, and No. 4). Also, objects intentionally hidden inside the main tank modify liquid volume measurements depending on the number of pieces (No. 5 and No. 6), while surrounding humidity can block the measure (No. 7). The ultrasound depth sensor measurements also change incorrectly when the tanks are hit with different intensities (No. 13, No. 14, and No. 15). Some examples of signal alterations are represented in Fig. 3, Fig. 4, Fig. 5.

**Table 1:** List of log files that compose the dataset.

**No.**	**Situation**	**Affected component**	**Operational scenario**	**Duration (hh:mm:ss)**	**Size**
1	Normal	None	Normal	02:01:47	7.3 MB
2	Plastic bag	Ultrasound sensor	Accident/Sabotage	00:33:20	4.2 MB
3	Blocked measure 1	Ultrasound sensor	Breakdown/Sabotage	00:00:25	74 KB
4	Blocked measure 2	Ultrasound sensor	Breakdown/Sabotage	00:00:17	48 KB
5	Floating objects in main tank (2 objects)	Ultrasound sensor	Accident/Sabotage	00:01:35	272 KB
6	Floating objects in main tank (7 objects)	Ultrasound sensor	Accident/Sabotage	00:01:22	234 KB
7	Humidity	Ultrasound sensor	Breakdown	00:00:18	52 KB
8	Discrete sensor failure	Discrete sensor 1	Breakdown	00:13:55	1.8 MB
9	Discrete sensor failure	Discrete sensor 2	Breakdown	00:03:40	610 KB
10	Denial of service attack	Network	Cyber-attack	00:01:37	102 KB
11	Spoofing	Network	Cyber-attack	00:34:33	3.2 MB
12	Wrong connection	Network	Breakdown/Sabotage	00:15:33	1.7 MB
13	Person hitting the tanks (low intensity)	Whole subsystem	Sabotage	00:00:39	112 KB
14	Person hitting the tanks (medium intensity)	Whole subsystem	Sabotage	00:00:32	91 KB
15	Person hitting the tanks (high intensity)	Whole subsystem	Sabotage	00:00:33	95 KB

Additionally, two of the discrete sensors (1 and 2) were disrupted by keeping each one at a blocked position, i.e. up when the liquid has Not reached that level yet (No. 8) and pushing randomly down once liquid overflowed it (No. 9), leaving the tank almost empty or filling up to the security aperture, respectively. Network intrusions were carried out making use of the Modbus Penetration Testing Framework, Smod,^1^ to execute a denial of service attack (No. 10) and a spoofing attack (No. 11). Finally, aNomalies can also be the result of unintentional human errors as a wrong system connection (No. 12) and more generally incorrect maintenance. Technical data sheets of the ultrasound sensor and the PLC, the network schema, the transmitted information between components, a script written in Python to read and display files, and additional details are provided with the dataset.

## Footnotes

1 Smod project. Available in: https://github.com/enddo/smod.
