# Supplementary material for: Dataset of anomalies and malicious acts in a cyber-physical subsystem
Source: Data Brief. 2017 Jul 20;14:186–91. doi: 10.1016/j.dib.2017.07.038 (PMC5536820; doi:10.1016/j.dib.2017.07.038)
Supplement: Supplementary file 1 — Supplementary material [file mmc1.pdf]

Conflict of Interest statement

Data in Brief

Manuscript Number: DIB-D-17-00383

Manuscript Title: Dataset of anomalies and malicious acts in a cyber-physical subsystem

Authors: J Pedro Merino Laso, David Brosset, and John Puentes

Conflict of interest: None declared
