# Supplementary material for: Dataset of anomalies and malicious acts in a cyber-physical subsystem
Source: Data Brief. 2017 Jul 20;14:186–91. doi: 10.1016/j.dib.2017.07.038 (PMC5536820; doi:10.1016/j.dib.2017.07.038)
Supplement: Supplementary file 2 [file mmc2.zip › dataset/index.html]

 


 Tanks Dataset 


- Introduction
- Description
- How to use it
- Files
- Datasheets
- Scripts
- Authors
 


# Dataset of anomalies and malicious acts in a cyber-physical subsystem.

Given interconnectedness, technology fragmentation and vulnerability, cyber-physical systems
are increasingly prone to faulty operation. Problems can be induced by anomalies of system
components, or external malicious acts liye sabotages and cyber-attacys. Although cyber-
physical systems are critical and ubiquitous, i.e. transport, industry, and smart cities, datasets to
investigate and compare faulty operation detection and characterization are not available.

We present a dataset produced using a supervisory control and data acquisition system formed by
two tanks - liquid containers for fuel or water –, two pumps, one ultrasound sensor, and four
discrete liquid level sensors, as well as a programmable logic controller. Sets of temporal series
representing normal operation, anomalies, and malicious acts observed in this cyber-physical
subsystem were collected in 15 files of different duration.


---

### The platform

Two tanks of different volumes, one ultrasound depth sensor, four discrete sensors, two pumps, and
an alarm indicator form the used cyber-physical subsystem. It is controlled by one computer
through a PLC connected to a control and monitoring network, and can function as water or fuel storage
and distribution device.

### Dataset

A computer connected to the control and monitoring network acquired the time series of the cyber-
physical subsystem. Working on automatic mode, log files’ values were recorded after sending a
periodic read request to each register of the PLC applying a scan time of 0.1 seconds. Collected data for
all scenarios were stored in CSV files.

# Description.

### Platform Description

An ultrasound depth sensor – model XX918A3F1M12 made by Telemecanique and largely used in
industrial systems for measuring distances or presence detection is installed on top of the main tank,
to measure the distance from the sensor to the liquid surface.

After being filled by pomp 2 from a recovery tank, the second tank transfers liquid to the main tank
when pump 1 is activated. Constant liquid consumption is generated by an opening in the bottom of
the main tank. To avoid overflowing, both tanks have a security aperture at the top.

A PLC fabricated by Schneider Electric – model TWDLCAE40DRF with an expansion analog input
module TM2AMI2HT – is commanded in two different ways: a touch screen or a remote system
connected to the network. Components of this cyber-physical platform communicate using the
Modbus/IP [A] network protocol and a generic switch for connections.

Furthermore, the platform can function according to either manual or automatic modes. In manual
mode, activation is carried out from the touch screen or from a remote system. In automatic mode,
the pump that fills the second tank is activated when its liquid volume goes below 1.25 L (considered
as low level) and stops when a volume of 9 L (considered as high level) is reached. Similarly, the
pump that fills the main tank is triggered when the ultrasound sensor measures less than 2.1 L (step
3 000) and it stays turned on until a level of 6.3 L (step 9 000) is reached. An example of these signals
is illustrated in Fig. 2. The pumps and discrete sensor states are: “on” (1.0) and “off” (0.0).

### Schema of the network:

### Acquisition protocol

A computer connected to the control and monitoring network acquired the time series of the
cyber-physical subsystem. Working on automatic mode, log files’ values were recorded after sending
a periodic read request to each register of the PLC applying a scan time of 0.1 seconds. Collected
data for all scenarios were stored in CSV files.

### Structure of the data log registers

Three registers of the PLC (Table A), identified from 2 to 4, provided output data. Register 2
provided the bits that indicate the binary state of the four discrete sensors, identified as DS i . Register
3 contained the binary state of the two pumps identified as P j . Register 4 stored the value of the
ultrasound sensor measure.

Data read on the PLC were stored in CVS log files following the same order of items for all the files
of the dataset, namely: Time stamp, register, and value. The time stamp corresponds to: date and
time using the format dd/mm/yyyy hh:mm:ss.sss. A script in Python complements the dataset to
ease its use. This code reads the CSV files, allocates time series in the corresponding vectors, and
permits the visualization of the signals.

---


---

You can find more detailed information on the data in brief article.

---

# How to use it.

This work is licensed under a
Creative Commons Attribution-ShareAlike 4.0 International License. You can find detailled information bellow.

### You are free to:

**Share**  — copy and redistribute the material in any medium or format.

**Adapt**  — remix, transform, and build upon the material for any purpose, even commercially.

The licensor cannot revoke these freedoms as long as you follow the license terms.

### Under the following terms:

**Attribution** — You must give appropriate credit, provide a link to the license, and indicate if changes were made. You may do so in any reasonable manner, but not in any way that suggests the licensor endorses you or your use.

**ShareAlike** — If you remix, transform, or build upon the material, you must distribute your contributions under the same license as the original.

**No additional restrictions** — You may not apply legal terms or technological measures that legally restrict others from doing anything the license permits.

To attribute this work to the authors you should cite this article.

---

# Files.

| Scenario | Affected sub-system | Type of event | Duration (hh:mm:ss) | File | Size |
| --- | --- | --- | --- | --- | --- |
| Normal | None | Normal | 02:01:47 | Download | 7.3 MB |
| Plastic bag | Ultrasounder sensor | Accident / Sabotage | 00:33:20 | Download | 4.2 MB |
| Blocked measure 1 | Ultrasounder sensor | Breakdown / Sabotage | 00:00:25 | Download | 74 KB |
| Blocked measure 2 | Ultrasounder sensor | Breakdown / Sabotage | 00:00:17 | Download | 48 KB |
| Floating objects in main tank (2 objects) | Ultrasounder sensor | Accident / Sabotage | 00:01:35 | Download | 272 KB |
| Floating objects in main tank (7 objects) | Ultrasounder sensor | Accident / Sabotage | 00:01:22 | Download | 234 KB |
| Humidity | Ultrasounder sensor | Breakdown | 00:00:18 | Download | 52 KB |
| Discrete sensor failure 1 | Discrete sensor 1 | Breakdown | 00:13:55 | Download | 1.8 MB |
| Discrete sensor failure 2 | Discrete sensors 2 | Breakdown | 00:03:40 | Download | 610 KB |
| Denial of Service attack | Network | Cyber-attack | 00:01:37 | Download | 102 KB |
| Spoofing | Network | Cyber-attack | 00:34:33 | Download | 3.2 MB |
| Wrong conection | Network | Breakdown / Sabotage | 00:15:33 | Download | 1.7 MB |
| Person hitting the tanks (low intensity) | Whole subsystem | Sabotage | 00:00:39 | Download | 112 KB |
| Person hitting the tanks (medium intensity) | Whole subsystem | Sabotage | 00:00:32 | Download | 91 KB |
| Person hitting the tanks (high intensity) | Whole subsystem | Sabotage | 00:00:33 | Download | 95 KB |

---

# Datasheets.

PLC Datasheet - Download 

Analog input module Datasheet - Download 

Ultrasounder Datasheet - Download

---

# Script.

This python script allows reading and plotting the log files to ease the utilization of the dataset.   
 Download

---

# Authors.

### Pedro Merino Laso a

Email:  pedro@merinolaso.eu   
Website:  www.merinolaso.eu   

### David Brosset ab

Email:  david.brosset@ecole-navale.fr   

### John Puentes ac

Email:  john.puentes@imt-atlantique.fr   
  
a) Chair of Naval Cyber Defense. École Navale - CC 600 F29240 Brest Cedex 9 France  
b) Naval Academy Research Institute École Navale - CC 600 F29240 Brest Cedex 9 France  
c) Dpt. Image et Traitement de l'Information. Institut Mines-Télécom Atlantique Bretagne Pays de la Loire; Lab-STICC UMR CNRS 6285 Équipe DECIDE, CS 83818 29238. Brest Cedex 3, France

---


- © Copyright 2014 CeeVee
- Design by Styleshout

 
