# Supplementary material for: Dataset of anomalies and malicious acts in a cyber-physical subsystem
Source: Data Brief. 2017 Jul 20;14:186–91. doi: 10.1016/j.dib.2017.07.038 (PMC5536820; doi:10.1016/j.dib.2017.07.038)
Supplement: Supplementary file 2 [file mmc2.zip › dataset/datasheets/twdlcae40drf-datasheet.pdf]

Product data sheet
Characteristics

TWDLCAE40DRF
compact PLC base Twido - 100..240 V AC supply - 24 I 24 V DC - 16 O

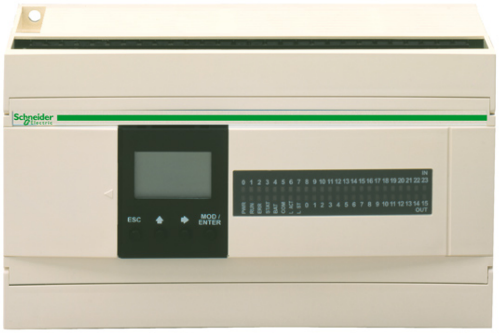

Main

Range of product	Twido
Product or component type	Compact base controller
Discrete I/O number	40
Discrete input number	24
Discrete input voltage	24 V
Discrete input voltage type	DC
Discrete output number	2 transistor<br>14 relay
Number of I/O expansion module	7
[Us] rated supply voltage	100...240 V AC
Use of slot	Memory cartridge
Data backed up	Internal RAM external battery TSXPLP01 3 years
Integrated connection type	Ethernet TCP/IP RJ45 10/100 Mbit/s 1 twisted pair transparent ready class A10<br>Non isolated serial link mini DIN Modbus/character mode master/slave RTU/ASCII RS485 half duplex 38,4 kbit/s<br>Power supply<br>Serial link interface adaptor RS232C/RS485
Complementary function	PID<br>Event processing

Complementary

Concept	Transparent Ready
Discrete input logic	Sink or source
Input voltage limits	20.4...26.4 V
Discrete input current	7 mA I0.2 to I0.5<br>7 mA I0.8 to I0.23<br>11 mA I0.0 to I0.1<br>11 mA I0.6 to I0.7
Input impedance	2100 Ohm I0.0 to I0.1<br>2100 Ohm I0.6 to I0.7<br>3400 Ohm I0.2 to I0.5<br>3400 Ohm I0.8 to I0.23
Filter time	35 µs + programmed filter time for I0.0 to I0.5 at state 1<br>40 µs + programmed filter time for I0.0 to I0.5 at state 0<br>40 µs + programmed filter time for I0.6 to I0.23 at state 1<br>150 µs + programmed filter time for I0.6 to I0.23 at state 0
Insulation between channel and internal logic	1500 Vrms for 1 minute
Insulation resistance between channel	None
Minimum load	0.1 mA
Contact resistance	≤ 30000 µOhm
Load current	2 A 240 V AC resistive 30 cyc/mn relay outputs<br>2 A 30 V DC resistive 30 cyc/mn relay outputs<br>2 A 240 V AC inductive 30 cyc/mn relay outputs<br>2 A 30 V DC inductive 30 cyc/mn relay outputs
Mechanical durability	≥ 20000000 cycles relay outputs
Electrical durability	≥ 100000 cycles relay outputs

The information provided in this documentation contains general descriptions and/or technical characteristics of the performance of the products contained herein.
This documentation is not intended as a substitute for and is not to be used for determining suitability or reliability of these products for specific user applications.
It is the duty of any such user or integrator to perform the appropriate and complete risk analysis, evaluation and testing of the products with respect to the relevant specific application or use thereof.
Neither Schneider Electric Industries SAS nor any of its affiliates or subsidiaries shall be responsible or liable for misuse of the information contained herein.

Current consumption	5 mA 24 V DC at state 0<br>90 mA 5 V DC at state 1<br>128 mA 24 V DC at state 1<br>128 mA 24 V DC state 1 + input ON<br>170 mA 5 V DC at state 0<br>240 mA 5 V DC state 1 + input ON
I/O connection	Non-removable screw terminal block
Input/Output number	≤ 152 removable screw terminal block with I/O expansion module<br>≤ 208 spring terminal block with I/O expansion module<br>≤ 264 HE-10 connector with I/O expansion module
Network frequency	50/60 Hz
Supply voltage limits	85...264 V
Network frequency limits	47...63 Hz
Power supply output current	0.4 A 24 V DC sensors
Power supply input current	790 mA
Inrush current	≤ 35 A
Protection type	Power protection internal fuse
Power consumption in VA	65 VA 100 V<br>77 VA 264 V
Insulation resistance	> 10 MOhm at 500 V, between supply and earth terminals<br>> 10 MOhm at 500 V, between I/O and earth terminals
Program memory	3000 instructions
Exact time for 1 K instruction	1 ms
System overhead	0.5 ms
Memory description	Internal RAM 256 internal bits, no floating, no trigonometrical<br>Internal RAM 3000 internal words, no floating, no trigonometrical<br>Internal RAM 128 timers, no floating, no trigonometrical<br>Internal RAM 128 counters, no floating, no trigonometrical<br>Internal RAM double words, no floating, no trigonometrical<br>Internal RAM floating, trigonometrical
Free slots	1
Realtime clock	With <= 30 s/month 30 days
Port Ethernet	10BASE-T/100BASE-TX
Communication service	BOOTP client Ethernet TCP/IP<br>Modbus messaging Ethernet TCP/IP
Positioning functions	PWM/PLS 2 7 kHz
Counting input number	2 20000 Hz 32 bits<br>4 5000 Hz 16 bits
Analogue adjustment points	1 point adjustable from 0...1023<br>1 point adjustable from 0 to 511 points
Marking	CE
Status LED	1 LED green PWR<br>1 LED green RUN<br>1 LED red module error (ERR)<br>1 LED user pilot light (STAT)<br>1 LED Ethernet status (LAN ST)<br>1 LED 10 or 100 Mbit/s rate (LACT)<br>1 LED per channel green I/O status
Product weight	0.525 kg

Environment

Immunity to microbreaks	10 ms
Dielectric strength	1500 V for 1 minute, between supply and earth terminals<br>1500 V for 1 minute, between I/O and earth terminals
Product certifications	CSA<br>UL
Ambient air temperature for operation	0...55 °C
Ambient air temperature for storage	-25...70 °C
Relative humidity	30...95 % without condensation
IP degree of protection	IP20
Operating altitude	0...2000 m
Storage altitude	0...3000 m

Vibration resistance	0.075 mm 10...57 Hz 35 mm symmetrical DIN rail<br>1 gn 57...150 Hz 35 mm symmetrical DIN rail<br>1.6 mm 2...25 Hz plate or panel with fixing kit<br>4 gn 25...100 Hz plate or panel with fixing kit
Shock resistance	15 gn 11 ms
RoHS EUR conformity date	0932
RoHS EUR status	Compliant
