# Supplementary material for: Dataset of anomalies and malicious acts in a cyber-physical subsystem
Source: Data Brief. 2017 Jul 20;14:186–91. doi: 10.1016/j.dib.2017.07.038 (PMC5536820; doi:10.1016/j.dib.2017.07.038)
Supplement: Supplementary file 2 [file mmc2.zip › dataset/datasheets/TM2AMI2HT-datasheet.pdf]

Product datasheet
Characteristics

TM2AMI2HT

analog input module M238 - 2 inputs voltage/current high level- non differential

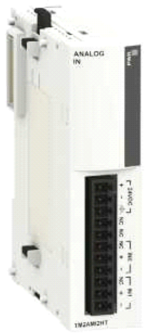

Main

Range of product	Modicon M238 logic controller
Product or component type	Analogue input module
Analogue input number	2
Input level	High level
Analogue input type	Current 4...20 mA non differential<br>Voltage 0...10 V non differential
Cross talk	<= 2 LSB

Complementary

Range compatibility	Advantys OTB<br>Twido
Analogue input resolution	12 bits
LSB value	2.5 mV voltage voltage<br>4.8 µA current current
Permissible continuous overload	13 V voltage<br>40 mA current
Input impedance	>= 1 MOhm voltage<br>10 Ohm current
Sampling duration	<= 10 ms
Acquisition period	10 ms per channel + 1 controller cycle time
Measurement error	+/- 0.2 % of full scale 25 °C
Temperature coefficient	+/-0.006 %FS/°C
Repeat accuracy	+/-0.5 %FS
Non-linearity	+/- 0.2 %FS
Total error	+/-1 %FS
Type of cable	Shielded cable
Insulation between channel and internal logic	Photocoupler
Supply	External supply
[Us] rated supply voltage	24 V DC
Supply voltage limits	20.4...28.8 V
Electrical connection	1 removable screw terminal block
Current consumption	40 mA 24 V DC external<br>50 mA 5 V DC internal
Product weight	0.085 kg

Environment

dielectric strength	500 V between the I/O and internal logic<br>500 V between the I/O and the external supply circuit
width	23.5 mm
depth	70 mm
height	90 mm

Offer Sustainability

Sustainable offer status	Green Premium product
RoHS (date code: YYWW)	Compliant - since 1039 - Schneider Electric declaration of conformity

The information provided in this documentation contains general descriptions and/or technical characteristics of the performance of the products contained herein.
This documentation is not intended as a substitute for and is not to be used for determining suitability or reliability of these products for specific user applications.
It is the duty of any such user or integrator to perform the appropriate and complete risk analysis, evaluation and testing of the products with respect to the relevant specific application or use thereof.
Neither Schneider Electric Industries SAS nor any of its affiliates or subsidiaries shall be responsible or liable for misuse of the information contained herein.

REACh	Reference not containing SVHC above the threshold
Product environmental profile	Available
Product end of life instructions	Available

Contractual warranty

Warranty period	18 months

Analog Input Module (2-channel, Voltage/Current)

Dimensions

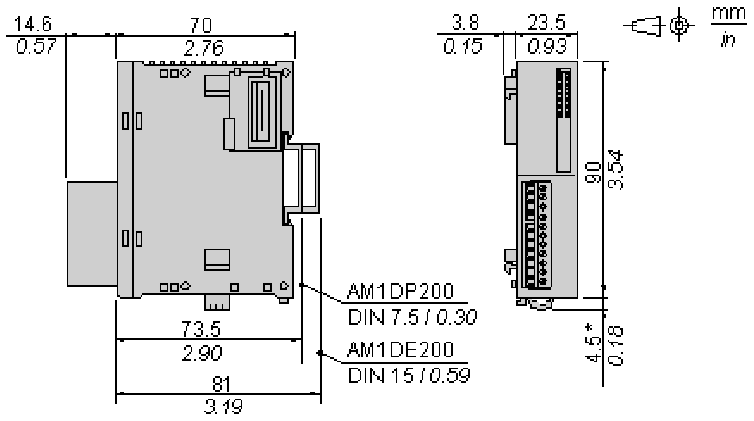

NOTE: * 8.5 mm *(0.33 in)* when the clip-on lock is pulled out.

DIN Rail Mounting

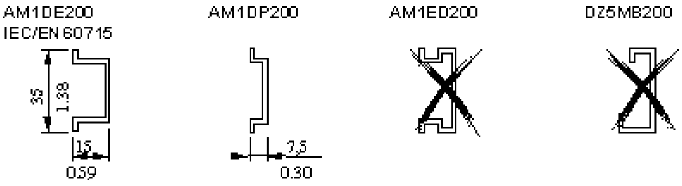

Rail depth	Catalogue part number
15 mm (*0.59 in.*)	AM1DE200
7,5 mm (*0.30 in.*)	AM1DP200

NOTE: Do not use AM1ED200 and DZ5MB200

Module Mounting on a Panel Surface

Mounting Hole Layout

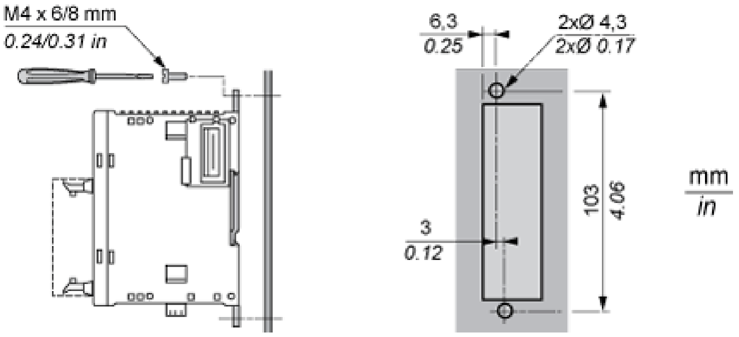

Wiring Requirements

Cable Types and Wire Sizes for Removable Screw Terminal Block

9 0.35 mm in.							
mm 2	0,14...1,5	0,25...0,5	0,25...1,5	0,14...0,5	0,14...0,75	0,25...0,34	0,5
AWG	26...16	24...20	24...16	26...20	26...18	24...22	20

Analog Input Module (2-channel, Voltage/Current)

Wiring Diagram

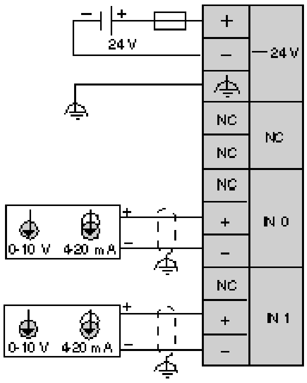

The (-) poles of inputs IN0 and IN1 are connected internally.
