# Supplementary material for: Dataset of anomalies and malicious acts in a cyber-physical subsystem
Source: Data Brief. 2017 Jul 20;14:186–91. doi: 10.1016/j.dib.2017.07.038 (PMC5536820; doi:10.1016/j.dib.2017.07.038)
Supplement: Supplementary file 2 [file mmc2.zip › dataset/datasheets/twdlcae40drf-datasheet-es.pdf]

Ficha de producto
Carácterísticas

TWDLCAE40DRF

CPU compacta Twido - alimentación 100-240 V CA - 24 E 24 V CC - 16 S

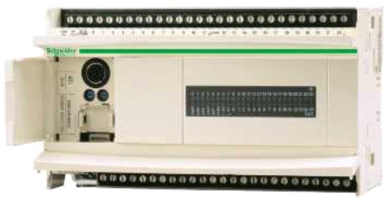

Principal

Gama de producto	Twido
Tipo de producto o componente	Controlador de base compacta
Concepto	Transparent Ready
Número de E/S digitales	40
Número de entrada digital	24
Tensión de entrada digital	24 V
Tipo de voltaje entrada discreto	CC
Número de salidas discretas	14 para relé<br>2 para transistor
[Us] tensión de alimentación nominal	100...240 V AC
Nº mód. expansión de E/S	7
Uso de la ranura	Cartucho mem
Orejetas terminales de anillo	RAM interna (batería externa TSXPLP01) 3 años
Tipo de conexión integrada	Ethernet TCP/IP RJ45, , 10/100 Mbit/s, 1 par trenzad Transparent Ready clase A10<br>Alimentación<br>Adaptador interfaz enlace serie (RS232C/RS485)<br>Enlace serie sin aislar mini DIN, Modbus/character mode maestro/esclavo RTU/ASCII (RS485) dúplex med., 38,4 kbit/s
Función complementaria	Procesamiento de evento<br>PID

Complementario

Lógica de entrada digital	Recep. o fuent.
Límites de tensión de entrada	20.4...26.4 V
Corriente de entrada discreta	11 mA para I0.0 a I0.1<br>11 mA para I0.6 a I0.7<br>7 mA para I0.2 a I0.5<br>7 mA para I0.8 a I0.23
Tapa de conexiones trasero	2100 Ohm para I0.0 a I0.1<br>2100 Ohm para I0.6 a I0.7<br>3400 Ohm para I0.2 a I0.5<br>3400 Ohm para I0.8 a I0.23
Cable troncal	150 µs + tiempo de filtro programado para I0.6 a I0.23 en estado 0<br>35 µs + tiempo de filtro programado para I0.0 a I0.5 en estado 1<br>40 µs + tiempo de filtro programado para I0.0 a I0.5 en estado 0<br>40 µs + tiempo de filtro programado para I0.6 a I0.23 en estado 1
Aislamiento entre canal y lógica interna	1500 Vrms para 1 minuto
Resistencia de aislamiento entre canal	Ninguno
Carga mínima	0.1 mA
Resistencia de los contactos	<= 30000 µOhm
2 abrazaderas	2 A en 240 V CA inductivo carg, ritmo funcion = 30 cyc/mn para salida del relé<br>2 A en 240 V CA resistivo carg, ritmo funcion = 30 cyc/mn para salida del relé<br>2 A en 30 V CC inductivo carg, ritmo funcion = 30 cyc/mn para salida del relé<br>2 A en 30 V CC resistivo carg, ritmo funcion = 30 cyc/mn para salida del relé
Durabilidad mecánica	>= 20000000 ciclos para salida del relé
Durabilidad eléctrica	>= 100000 ciclos para salida del relé
Consumo de corriente	128 mA en 24 V CC en estado 1<br>128 mA en 24 V CC estado 1 + entrada ON<br>170 mA en 5 V CC en estado 0<br>240 mA en 5 V CC estado 1 + entrada ON<br>5 mA en 24 V CC en estado 0<br>90 mA en 5 V CC en estado 1

La información incluida en esta documentación contiene descripciones generales y/o características técnicas de los productos. Esta documentación no pretende sustituir ni ser utilizada como herramienta garantizada de creación de especificaciones específicas para usuarios finales. Es responsabilidad del usuario final o integrador de realizar un apropiado análisis de riesgos, evaluación y testeo de los productos con respecto una aplicación específica. Schneider Electric Industries SAS o cualquiera de sus empresas subsidiarias o comercializadoras no se hacen responsables de una posible mala interpretación o uso de la documentación incluida en este documento

Conexión de E/S	Bornero de tornillo no extraíble
Refuerzo kit	<= 152 con bornero de tornillo extraíble con módulo de expansión de E/S<br><= 208 con bornero de resorte con módulo de expansión de E/S<br><= 264 con conector HE-10 con módulo de expansión de E/S
Frecuencia de red	50/60 Hz
Límites tensión alimentación	85...264 V
Límites de Frecuencia asignada de empleo	47...63 Hz
Corriente de salida fuente de alimentación	0.4 A para dectector 24 V CC
Corriente de entrada de alimentación	790 mA
Corriente de entrada	<= 35 A
Tipo de protección	Protección de alimentac. con fusible interno
Consumo de potencia en VA	65 VA en 100 V<br>77 VA en 264 V
De la resistencia de aislamiento	> 10 MOhm a 500 V, entre E/S y terminales a tierra<br>> 10 MOhm a 500 V, entre suministro y terminales a tierra
Memoria de programa	3000 instrucciones
Hora exacta para 1 Kinstruction	1 ms
Línea aérea del sistema	0.5 ms
Descripción de memoria	RAM interna, 128 contadores, no flotantes, no trigonométrico<br>RAM interna, 128 temporizadores, no flotantes, no trigonométrico<br>RAM interna, 256 bitios internos, no flotantes, no trigonométricos<br>RAM interna, 3000 palabras internas, no flotantes, no trigonométrico<br>RAM interna, palabras dobles, no flotantes, no trigonométrico<br>RAM interna, flotante, trigonométrico
Ranuras libres	1
Reloj en tiempo real	Donde, pila: <= 30 s/mes, tiempo funcion: 30 días
Puerto Ethernet	10BASE-T/100BASE-TX
Servicio de comunicación	Cliente BOOTP Ethernet TCP/IP<br>Mensajería Modbus Ethernet TCP/IP
Funciones de posicionamiento	PWM/PLS 2 canal(es) en 7 kHz
Número de entrada de contaje	2 canal(es) en 20000 Hz 32 bits<br>4 canal(es) en 5000 Hz 16 bits
Puntos de ajuste analógicos	1 punto ajustable de 0 a 1.023<br>1 punto ajustable de 0 a 511
LED de estado	1 LED para vel. 10 ó 100 Mbit/s (LACT)<br>1 LED para estado Ethernet (LAN ST)<br>1 LED para luz piloto usuario (STAT)<br>1 LED verde para PWR<br>1 LED verde para RUN<br>1 LED por canal verde para estado E/S<br>1 LED rojo para error de módulo (ERR)
Peso del producto	0.525 kg

Medioambiente

inmunidad a microcortes	10 ms
fuerza dialéctrica	1500 V para 1 minuto, entre E/S y terminales a tierra<br>1500 V para 1 minuto, entre suministro y terminales a tierra
certificaciones	CSA<br>UL
marca	CE
temperatura ambiente de trabajo	0...55 °C
temperatura ambiente de almacenamiento	-25...70 °C
humedad relativa	30...95 % sin condensación
grado IP	IP20
altitud máxima de funcionamiento	0...2000 m
altitud de almacenamiento	0...3000 m
resistencia a las vibraciones	0.075 mm, 10...57 Hz montaje el: perfil DIN simétrico de 35 mm<br>1 gn, 57...150 Hz montaje el: perfil DIN simétrico de 35 mm<br>1.6 mm, 2...25 Hz montaje el: placa o panel con juego de fijación<br>4 gn, 25...100 Hz montaje el: placa o panel con juego de fijación
resistencia a los choques	15 gn para 11 ms

Contractual warranty

Warranty period	18 months

Dimensions

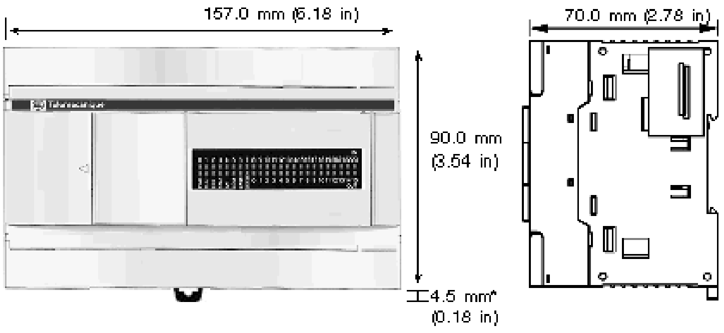

* 8.5 mm (0.33 in) when the clamp is pulled out.

Minimum Clearances for a Compact Base and Expansion I/O Modules

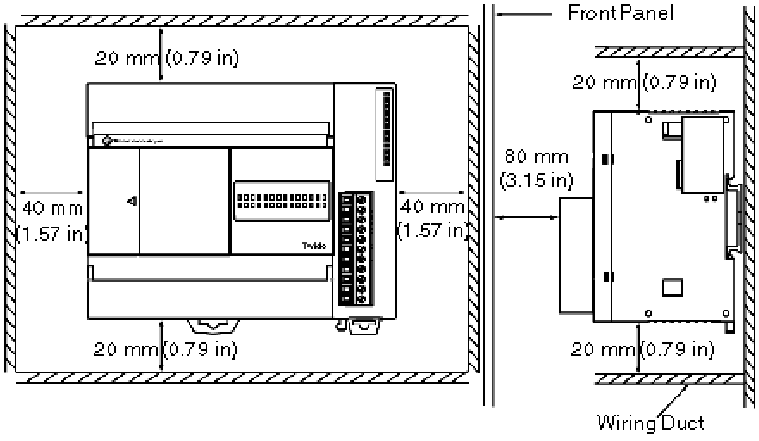

Mounting Hole Layout

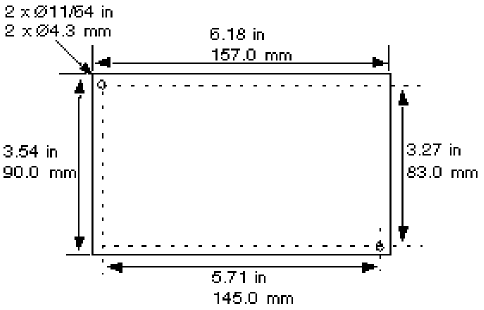

AC Power Supply Wiring Diagram

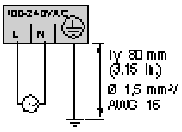

DC Source Inputs Wiring Diagrams

External Power

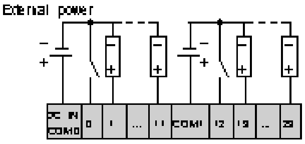

Internal Power

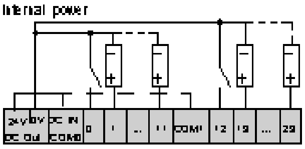

Max current: 400mA.

DC Sink Inputs Wiring Diagrams

External Power

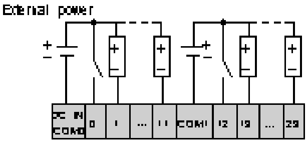

Internal Power

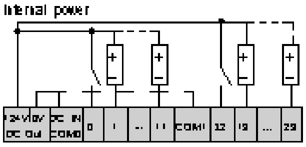

Max current: 400mA.

Relay and Transistor Outputs Wiring Diagram

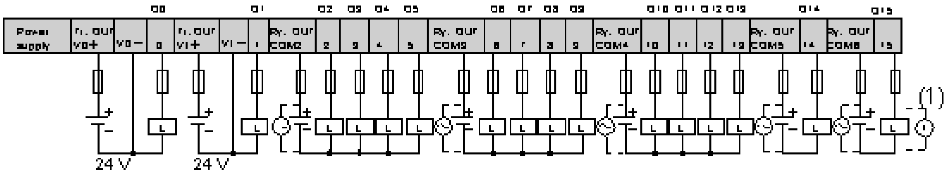

(1)

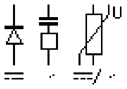

Performance Curves

I/O Usage Limits

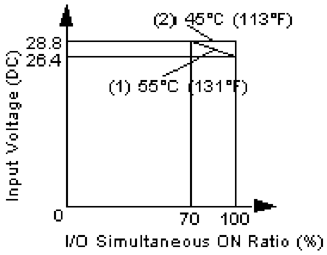

(1) Limit for TWDLC•AA16DRF, TWDLC•A24DRF, TWDLCA•40DRF and TWDLD•40DRF

(2) All compact bases
