# Supplementary material for: Dataset of anomalies and malicious acts in a cyber-physical subsystem
Source: Data Brief. 2017 Jul 20;14:186–91. doi: 10.1016/j.dib.2017.07.038 (PMC5536820; doi:10.1016/j.dib.2017.07.038)
Supplement: Supplementary file 2 [file mmc2.zip › dataset/datasheets/Schneider_Electric-XX918A3F1M12-datasheet.pdf]

Product datasheet
Characteristics

XX918A3F1M12

ultrasonic sensor cylindrical M18 - Sn 0.5 m - 0..10 V - M12 connector

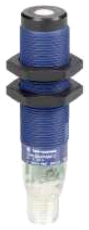

Main

Range of product	OsiSense XX
Sensor type	Ultrasonic sensor
Series name	General purpose
Sensor name	XX9
Sensor design	Cylindrical M18
Detection system	Diffuse
[Sn] nominal sensing distance	0.5 m adjustable with remote teach pushbutton
Material	Plastic
Type of output signal	Analogue
Wiring technique	4-wire
Analogue output function	0...10 V
[Us] rated supply voltage	12...24 V DC with reverse polarity protection
Electrical connection	Male connector M12 4 pins
[Sd] sensing range	0.051...0.508 m
Beam angle	6 °
IP degree of protection	IP67 conforming to IEC 60529

Complementary

Enclosure material	Valox
Front material	Epoxy
ISO thread	M18 x 1
Supply voltage limits	10...28 V DC
[Sa] assured operating distance	0.051...0.508 m (teach mode)
Blind zone	0...51 mm
Transmission frequency	300 kHz
Repeat accuracy	1.27 %
Deviation angle from 90° of object to be detected	-7...7 °
Minimum size of detected object	Cylinder diameter 1.6 mm
Status LED	1 LED (dual colour) for setting-up assistance<br>1 LED (green) for supply on<br>1 LED (yellow) for output state
Current consumption	40 mA
Maximum switching capacity	>= 1 kOhm with overload and short-circuit protection
Voltage drop	< 1 V
Setting-up	Slope selection using teach button
Delay first up	100 ms
Delay response	25 ms
Delay recovery	25 ms
Marking	CE
Threaded length	43 mm
CAD overall height	18 mm
CAD overall width	18 mm
CAD overall depth	79 mm
Product weight	0.033 kg

Environment

Standards	IEC 60947-5-2

The information provided in this documentation contains general descriptions and/or technical characteristics of the performance of the products contained herein.
This documentation is not intended as a substitute for and is not to be used for determining suitability or reliability of these products for specific user applications.
It is the duty of any such user or integrator to perform the appropriate and complete risk analysis, evaluation and testing of the products with respect to the relevant specific application or use thereof.
Neither Schneider Electric Industries SAS nor any of its affiliates or subsidiaries shall be responsible or liable for misuse of the information contained herein.

Ambient air temperature for operation	-20...65 °C
Ambient air temperature for storage	-40...80 °C
Vibration resistance	+/-1 mm conforming to IEC 60068-2-6 10...55 Hz
Shock resistance	30 gn in all 3 axes for 11 ms conforming to IEC 60068-2-27
Resistance to electrostatic discharge	8 kV level 4 conforming to IEC 61000-4-2
Resistance to electromagnetic fields	10 V/m level 3 conforming to IEC 61000-4-3
Resistance to fast transients	1 kV level 3 conforming to IEC 61000-4-4
