# Supplementary figures and images for: Dataset of anomalies and malicious acts in a cyber-physical subsystem
Source: Data Brief. 2017 Jul 20;14:186–91. doi: 10.1016/j.dib.2017.07.038 (PMC5536820; doi:10.1016/j.dib.2017.07.038)

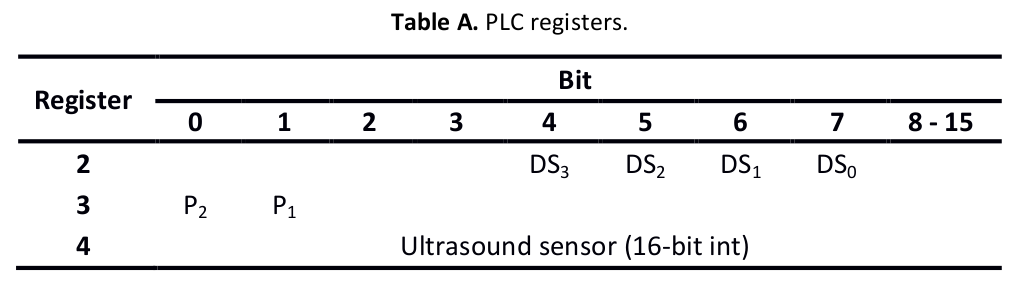

Supplement: Supplementary file 2 [file mmc2.zip › dataset/images/table_bits.png]

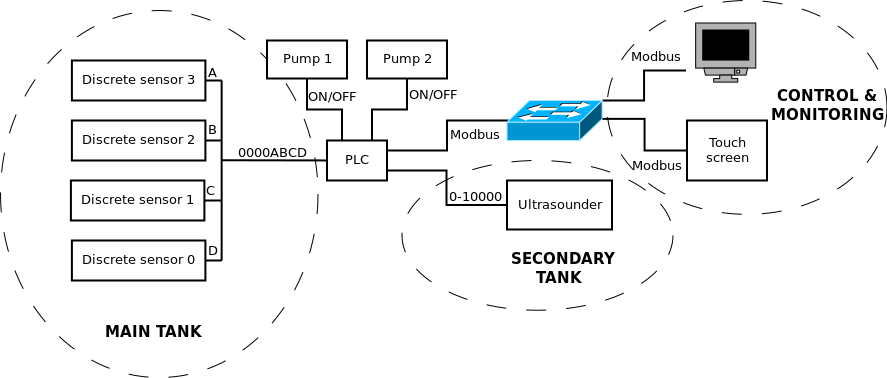

Supplement: Supplementary file 2 [file mmc2.zip › dataset/images/network_tanks.png]

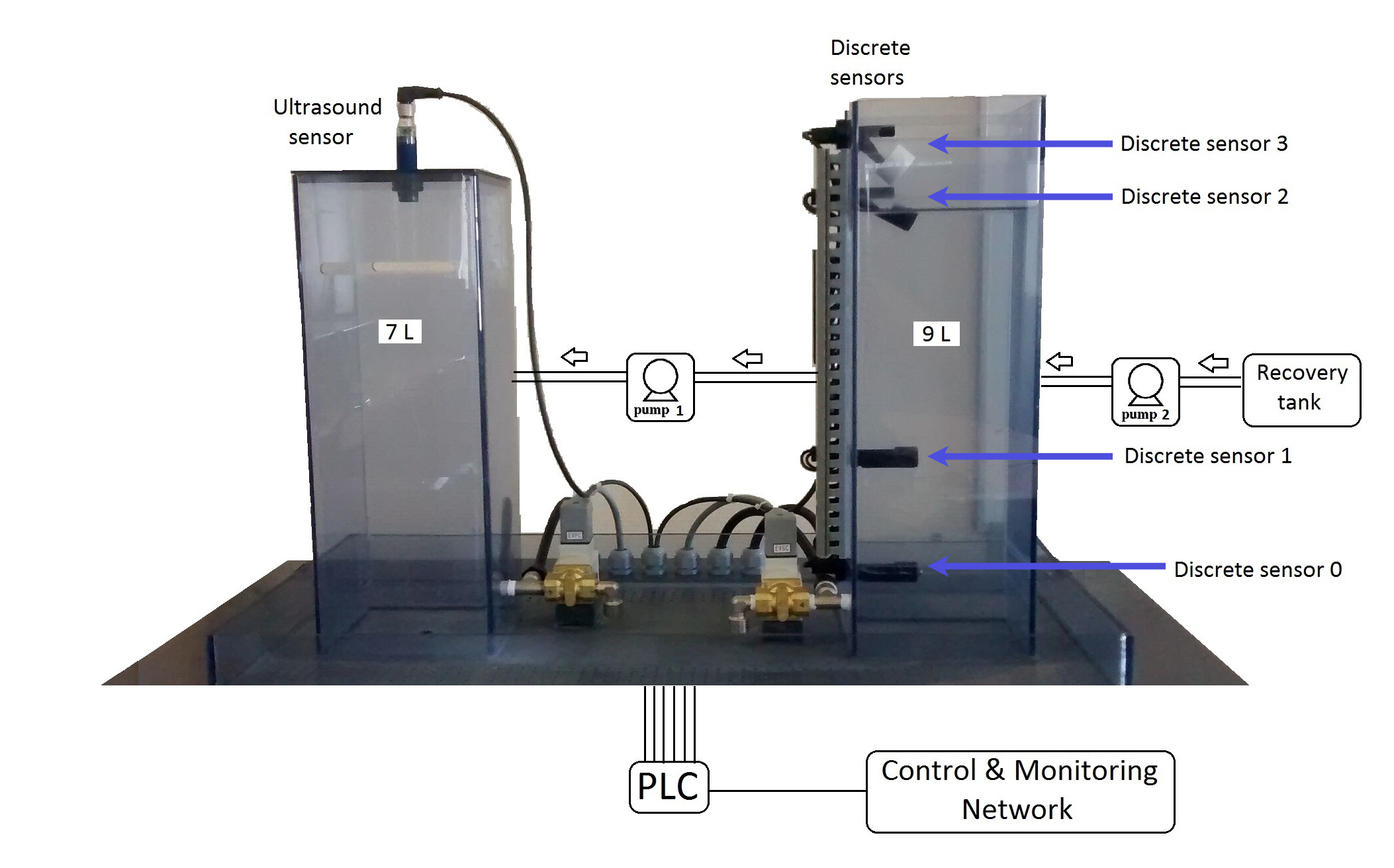

Supplement: Supplementary file 2 [file mmc2.zip › dataset/images/tanks.png]
